# Comparative phylogeography of two sympatric beeches in subtropical China: Species-specific geographic mosaic of lineages

**DOI:** 10.1002/ece3.829

**Published:** 2013-10-11

**Authors:** Zhi-Yong Zhang, Rong Wu, Qun Wang, Zhi-Rong Zhang, Jordi López-Pujol, Deng-Mei Fan, De-Zhu Li

**Affiliations:** 1 Laboratory of Subtropical Biodiversity, Jiangxi Agricultural UniversityNanchang, Jiangxi, 330045, China; 2 Key Laboratory of Biodiversity and Biogeography, Kunming Institute of Botany, Chinese Academy of SciencesKunming, Yunnan, 650204, China; 3 The Germplasm Bank of Wild Species in Southwest China, Kunming Institute of Botany, Chinese Academy of SciencesKunming, Yunnan, 650204, China; 4 Laboratori de Botànica, Facultat de Farmàcia, Universitat de BarcelonaAvda, Joan XXIII s/n, Barcelona, Catalonia, 08028, Spain

**Keywords:** *atp*I-*atp*H, chloroplast DNA, comparative phylogeography, *Fagus longipetiolata*, *Fagus lucida*, *ndh*J-*trn*F, subtropical China

## Abstract

In subtropical China, large-scale phylogeographic comparisons among multiple sympatric plants with similar ecological preferences are scarce, making generalizations about common response to historical events necessarily tentative. A phylogeographic comparison of two sympatric Chinese beeches (*Fagus lucida* and *F. longipetiolata*, 21 and 28 populations, respectively) was conducted to test whether they have responded to historical events in a concerted fashion and to determine whether their phylogeographic structure is exclusively due to Quaternary events or it is also associated with pre-Quaternary events. Twenty-three haplotypes were recovered for *F. lucida* and *F. longipetiolata* (14 each one and five shared). Both species exhibited a species-specific mosaic distribution of haplotypes, with many of them being range-restricted and even private to populations. The two beeches had comparable total haplotype diversity but *F. lucida* had much higher within-population diversity than *F. longipetiolata*. Molecular dating showed that the time to most recent common ancestor of all haplotypes was 6.36 Ma, with most haplotypes differentiating during the Quaternary. [Correction added on 14 October 2013, after first online publication: the timeunit has been corrected to ‘6.36’.] Our results support a late Miocene origin and southwards colonization of Chinese beeches when the aridity in Central Asia intensified and the monsoon climate began to dominate the East Asia. During the Quaternary, long-term isolation in subtropical mountains of China coupled with limited gene flow would have lead to the current species-specific mosaic distribution of lineages.

## Introduction

China harbors some of the most important global Quaternary refugia for lineages that evolved prior to the late Neogene and Quaternary glaciations (Axelrod et al. 1996), this being one of the main reasons for explaining the exceptionally high number of plants for this country (over 30,000 species; López-Pujol et al. 2011). Subtropical China, ranging between the Qingling Mountains–Huai River line (at *ca*. 34°N) and the tropical south (≤22°N) and being characterized by a mild monsoon climate and a complex topography, is particularly rich in ancient lineages (e.g., *Cathaya*, *Davidia*, *Dipteronia, Glyptostrobus, Metasequoia*, *Pseudolarix*, etc., Axelrod et al. 1996). Aimed to achieve a better understanding of the evolutionary history of this rich flora and also to inform conservation policy and management, phylogeographic studies have become one of the major research fields in evolutionary biology in China during recent years (see reviews in Qiu et al. 2011 and Liu et al. 2012). Deeply influenced by the concept of Quaternary glacial refugia and postglacial recolonization, most of these studies quote the Quaternary climatic cycles as the main factor in shaping population divergence; however, they rarely contain any time calibration to support this hypothesis (Qiu et al. 2011). Since the onset of the Neogene, the uplift of Himalayan–Tibetan Plateau (began about 50 Ma but significant increases in altitude occurred about 10–8 Ma or more recently), the formation of Asian monsoons (began about 9–8 Ma, continued intensification of the east Asian summer and winter monsoons about 3.6–2.6 Ma, increased variability and continued strengthening of the east Asian winter monsoon since about 2.6 Ma), and the aridification in Central Asia (the onset since 22 Ma, two peaks at 15–13 Ma and 8–7 Ma, and major increase after about 3.5 Ma) have profoundly influenced the geomorphology and climate of East Asia (An et al. 2001; Guo et al. 2002). These processes might be, thus, also candidates to explain the patterns of genetic diversity seen today (e.g., Yuan et al. 2008; Qi et al. 2012; Yan et al. 2013 and references therein), particularly for relict taxa. However, these historical events have been systematically omitted in the phylogeographic literature for plants in subtropical China.

Codistributed taxa with comparable ecological preferences and life-history traits may be similarly impacted by historical processes and thus share a congruent phylogeographic pattern (Garrick et al. 2008). Inferences from comparative phylogeographic studies can help in setting conservation priorities by identifying and delimiting areas with singular evolutionary histories and are useful for predicting how climate change will genetically, demographically, and spatially influence regional biodiversity (Moritz and Faith 1998; Hickerson et al. 2010). For plant species in subtropical China, a congruent pattern of range fragmentation and population isolation with limited or no spatial demographic expansion is emerging (Qiu et al. 2011; Lei et al. 2012; Liu et al. 2012). However, large-scale phylogeographic comparisons among multiple sympatric plants with similar ecological preferences are still scarce (Qiu et al. 2011), making generalizations about common response to historical events necessarily tentative. Because the rich flora of subtropical China is threatened by numerous environmental problems—mainly associated with high population density and a rapid economic development (Weber and Li 2008)—, comparative phylogeographic studies are urgently needed to identify areas of high priority for plant persistence and evolution (Moritz and Faith 1998; López-Pujol et al. 2011).

*Fagus* is a small ancient genus of *ca*. 9–10 monoecious tree species that occur in the Northern Hemisphere (Shen 1992; Denk 2003) and have an exceptionally well-studied fossil record extending back to the early Cenozoic (Denk and Grimm 2009). In North America, Europe and Japan, *Fagus* species are widespread in the temperate zone with a cool and moist climate. In China, however, they are absent in the deciduous forest of the temperate zone, but occur in mountainous areas in the moist subtropical zone, south of 34°N (Liu et al. 2003; Fang and Lechowicz 2006; Guo and Werger 2010). Ecological studies suggest that the prevailing Pacific monsoon, the low precipitation at Chinese northern latitudes, and the complex topography of southern China could be the responsible factors of their occurrence within subtropical mountains and their absence from northern China (Fang and Lechowicz 2006; Guo and Werger 2010).

**Figure 1:**
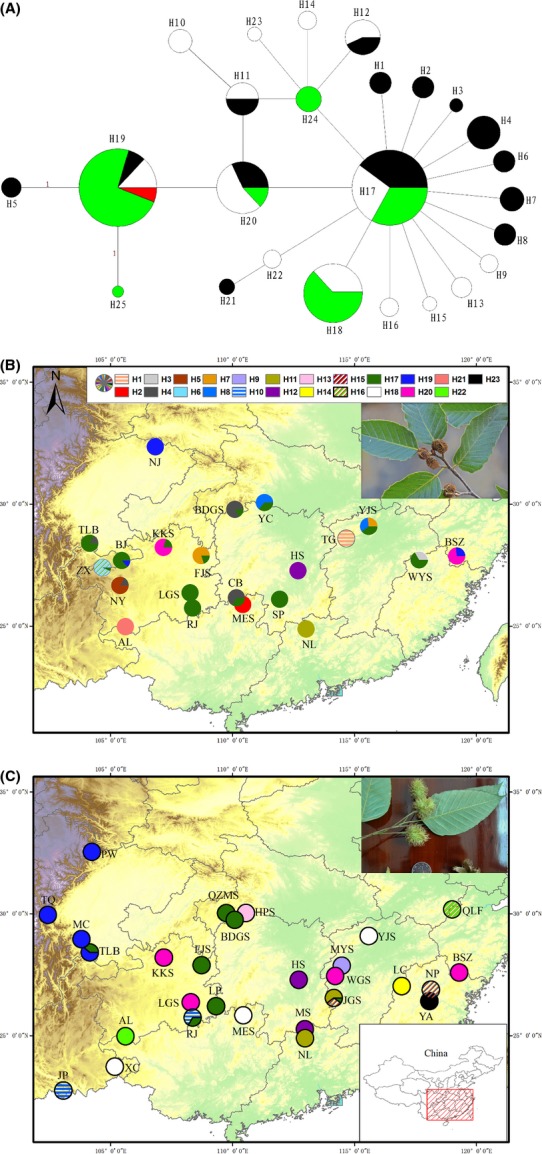
(A) Haplotype network of all four Chinese beeches; the black, white, green, and red colors represent *F. lucida*, *F. longipetiolata*, *F. engleriana,* and *F. hayatae*, respectively; (B) and (C) haplotype distribution in the studied populations of *Fagus lucida* and *F. longipetiolata*, respectively. The insets in (B) and (C) show the branches with capsules of each species.

There are four beech species distributed in subtropical China. Three species, *Fagus lucida*, *F. longipetiolata,* and *F. engleriana*, are widely distributed, whereas *F. hayatae* occurs only in scattered locations in mainland China and Taiwan (Huang et al. 1999). Among the three widely distributed beeches, *F. engleriana*, whose phylogeographic pattern was fully discussed in a previous study (Lei et al. 2012), is genetically distinct from the other beeches of China (as it is the only member belonging to subgen. *Engleriana*; Denk 2003; Denk et al. 2005; Denk and Grimm 2009) and mainly occurs in northern part of the Chinese beech range (i.e., in the coolest climate; Lei et al. 2012). The other two widespread beeches, *F. lucida* and *F. longipetiolata*, are largely sympatric across the whole subtropical China (Liu et al. 2003) with slightly different ecological preferences. *Fagus longipetiolata* always grows in more humid habitats and at lower altitudes than *Fagus lucida*, and coexistence in mixed stands happens occasionally (see Fig. S2). *Fagus lucida* is morphologically distinct from *F. longipetiolata*, because it bears smaller capsules (1–1.5 cm) with short peduncles (0.5–1.5 cm), tuberculate, and appresed bracts (1–2 mm), but *F. longipetiolata* bears larger capsules (2–2.5 cm) with long peduncles (1–10 cm), linear, and recurved bracts (*ca*. 7 mm for apical ones) (Huang et al. 1999; See also insets of Fig. 1B,C). Phylogenetically, *Fagus lucida* could be distinct from *F. longipetiolata,* as the former has a closer relationship to the Japanese *F. crenata* and the western Eurasian *F. sylvatica*, whereas the latter is related to *F. hayatae* and to the North American *F. grandifolia*, although they share some ITS variants (Denk et al. 2005; Grimm et al. 2007; Denk and Grimm 2009). Taking into account the shared distribution range and comparable ecological preferences and dispersal abilities between *F. lucida* and *F. longipetiolata*, one might suppose that the two beeches would have responded to historical events in a concerted fashion, which can be tested using phylogeographic methods. Furthermore, the long evolutionary history of *Fagus*, coupled with its exceptionally well-studied fossils, provides an excellent opportunity to reveal the impacts of pre-Quaternary events on phylogeographic patterns of these beech species by means of molecular dating.

By comparing the phylogeographic patterns of *F. lucida* and *F. longipetiolata* in subtropical China using two chloroplast genomic fragments*,* this study sought to address the following issues: (1) to discern whether the phylogeographic structure of *F. lucida* and *F. longipetiolata* is exclusively due to Quaternary events or it is also associated with pre-Quaternary events; (2) to know whether the two codistributed beeches have responded to historical events in a concerted fashion.

## Materials and Methods

### Population sampling and experimental procedure

As a result of extensive field surveys throughout the distribution range of *F. lucida* and *F. longipetiolata*, 276 individuals for each species were collected from 21 and 28 populations, respectively (Table 1, Fig. 1B,C). To avoid collecting genetically closely related individuals (such as full sibs or half full sibs), sampled trees were spaced by *ca*. 50–100 m. For the purpose of molecular dating, one species of three genera of Fagaceae, *Castanea*, *Lithocarpus,* and *Castanopsis*, were sampled (Table S1) as outgroups. All samples were desiccated in silica gel and stored at −20°C until being processed. Genomic DNA was extracted using a modified CTAB procedure. Two intergenic spacers (IGSs) of the chloroplast genome, *ndh*J-*trn*F and *atp*I-*atp*H, were amplified and sequenced, following the method described in Lei et al. (2012).

**Table 1:** Sample information, cpDNA haplotype frequency (number in bracket), and within-population haplotype diversity in *Fagus lucida* and *F. longipetiolata*. In italics, haplotypes specific to *F. lucida*; underlined, haplotypes specific to *F. longipetiolata*; in bold, shared haplotypes by the two species. H18 is in normal font because it is shared by *F. longipetiolata* and *F. engleriana*

Province	Population (abbr.)	Lat. (N)	Long. (E)	Alt. (m)	*N*	Hap. freq.	*h*_s_
	*Fagus lucida*						
Zhejiang	Baishanzu (BSZ)	27.88	119.18	1700	15	**H19** (4), **H20** (11)	0.419
Fujian	Wuyishan (WYS)	27.72	117.65	1400	15	*H3* (5), **H17** (10)	0.476
Jiangxi	Tonggu (TG)	28.6	114.66	1206	15	*H1* (15)	0
	Yunjushan (YJS)	29.1	115.58	698	15	*H7* (4), *H8* (5), **H17** (6)	0.705
Guangdong	Nanling (NL)	24.89	113.01	1660	15	**H11** (15)	0
Hubei	Yichang (YC)	30.07	111.3	1730	11	*H8* (7), **H17** (4)	0.509
Hunan	Chengbu (CB)	26.17	110.15	1580	15	*H4* (10), **H17** (5)	0.476
	Hengshan (HS)	27.28	112.67	1040	15	**H12** (15)	0
	Shuangpai (SP)	26.11	111.92	1376	4	**H17** (4)	0
	Badagongshan (BDGS)	29.79	110.09	1480	18	*H4* (15), **H17** (3)	0.294
Guangxi	Maoershan (MES)	25.89	110.41	1546	15	*H2* (15)	0
Guizhou	Nayong (NY)	26.68	105.38	1840	12	*H4* (2), *H5* (10)	0.303
Sichuan	Nanjiang (NJ)	32.36	106.83	1586	14	**H19** (14)	0
	Bijie (BJ)	27.69	105.45	1690	15	**H17** (13), **H19** (2)	0.248
	Kuankuoshui (KKS)	28.24	107.16	1746	15	**H17** (3), **H20** (12)	0.343
	Anlong (AL)	24.99	105.6	1457	8	*H21* (8)	0
	Leigongshan (LGS)	26.38	108.25	1385	8	**H17** (8)	0
	Rongjiang (RJ)	25.75	108.36	1100	9	**H17** (9)	0
	Fanjingshan (FJS)	27.9	108.71	1554	15	*H7* (12), **H17** (3)	0.343
Yunnan	Zhengxiong (ZX)	27.4	104.66	2129	15	*H6* (14), **H17** (1)	0.133
	Tongluoba (TLB)	28.4	104.14	1617	12	*H4* (2), **H17** (10)	0.303
	Summary				276		
	*Fagus longipetiolata*							
Zhejiang	Qiangliangfeng (QLF)	30.19	118.99	1000	10	H16 (10)	0
Fujian	Yongan (YA)	26.43	118.06	931	6	H23(6)	0
Jiangxi	Yunjushan (YJS)	29.1	115.58	698	10	H18 (10)	0
	Mingyueshan (MYS)	27.58	114.27	1431	10	H9 (10)	0
	Wugongshan (WGS)	27.45	114.19	1055	10	**H20** (10)	0
	Jinggangshan (JGS)	26.56	114.14	878	10	**H11** (6), H15 (3), **H17** (1)	0.6
	Lichuan (LC)	27.04	116.93	720	10	H14 (10)	0
Guangdong	Nanling (NL)	24.9	112.96	1100	10	**H11** (10)	0
	Nanping (NP)	26.69	118.13	1300	2	H15 (2)	0
	Baishanzu (BSZ)	27.6	119.28	1120	10	**H20** (10)	0
Hubei	Qizimeishan (QZMS)	30.04	109.73	1421	12	**H17** (12)	0
Hunan	Hupingshan (HPS)	30.04	110.54	1460	10	H13 (10)	0
	Badagongshan (BDGS)	29.76	110.08	1360	11	**H17** (11)	0
	Hengshan (HS)	27.3	112.71	770	10	**H12** (10)	0
	Mangshan (MS)	24.97	112.96	1064	10	**H12** (10)	0
Guizhou	Fanjingshan (FJS)	27.9	108.72	1098	11	**H17** (11)	0
Sichun	Tianquan (TQ)	29.96	102.42	1395	10	**H19 (10)**	0
	Pingwu (PW)	32.55	104.22	1380	11	**H19 (11)**	0
	Muchuan (MC)	28.97	103.79	1152	5	**H19 (5)**	0
	Kuankuoshui (KKS)	28.21	107.17	1446	10	**H20** (10)	0
	Maoershan (MES)	25.84	110.43	1123	13	H18 (13)	0
	Liping (LP)	26.22	109.31	912	10	**H17** (10)	0
	Leigongshan (LGS)	26.38	108.28	1385	10	**H20** (10)	0
	Anlong (AL)	24.99	105.6	1457	10	H22 (10)	0
	Rongjiang (RJ)	25.75	108.36	1100	9	H10 (6), **H17** (3)	0.5
Yunnan	Xichou (XC)	23.74	105.16	1660	13	H18 (13)	0
	Jinping (JP)	22.76	103.05	1760	10	H10 (10)	0
	Tongluoba (TLB)	28.42	104.14	1617	13	**H17** (5), **H19** (8)	0.512
	Summary				276		

### Analyses of genetic diversity and genetic structure

Because chloroplast genome can be regarded as a single locus, the two fragments were pooled together and indels were treated as missing data. Haplotype distribution maps were constructed using ARCGIS 9.3 (Redlands, CA) for both beech species. Because sharing of haplotypes among woody congeneric species is common (see Discussion), a distribution map of shared haplotypes between *F. lucida* and *F. longipetiolata* was also drawn. To ease comparisons, a haplotype map of the cooccurring studied locations of the two beeches was also provided. Total genetic diversity (*h*_T_) and within-population diversity (*h*_S_) were calculated with HAPLONST (Pons and Petit 1996). To determine phylogenetic relationships among haplotypes, median-joining networks were constructed using NETWORK 4.6 (Bandelt et al. 1999). The haplotypes found in *F. engleriana* and *F. hayatae* (Lei et al. 2012) were also included in constructing haplotype network as well as in molecular dating analysis.

The genetic structure of *F. lucida* and *F. longipetiolata* was analyzed separately; the population NP of *F. longipetiolata* was excluded from the analyses of genetic structure because of its small sample size (*n* = 2). We first evaluated whether there is a phylogeographic signal in the haplotype distribution by comparing *G*_ST_ with *N*_ST_ using the software HAPLONST (Pons and Petit 1996). To explore the geographically and genetically distinguishable groups and the potential barriers between groups, we analyzed the populations using SAMOVA 1.0 (Dupanloup et al. 2002). Various SAMOVA were run, increasing the number of *K* groups until the percentage of explained variance among groups reached a limit (*K* = 2–20). In addition, an AMOVA analysis was also implemented to partition the genetic variance within and between species using ARLEQUIN 3.1 (Excoffier et al. 2005). Finally, to examine the effect of geographic distance on genetic structure and the relative contribution of gene flow and drift to genetic structure (Hutchison and Templeton 1999), isolation-by-distance (IBD) analyses were performed employing the software GENALEX 6 (Peakall and Smouse 2006).

### Demographic analyses

We first conducted mismatch analysis using DNASP (version 5.0, Librado and Rozas 2009) to test whether *F. lucida* and *F. longipetiolata* have undergone recent demographic population expansion events. Multimodal mismatch distributions of pairwise differences between individuals are expected for populations at demographic equilibrium with a relatively stable size over time, whereas unimodal distributions are expected for populations that have experienced recent demographic expansions (Harpending 1994). The goodness-of-fit of observed mismatch distributions to the theoretical distributions under a model of sudden expansion was tested with the raggedness index (*r*) (Rogers and Harpending 1992). The significance of the raggedness index (*r*) was obtained by examining the null distribution of 5000 coalescent simulations of these statistics. Small raggedness values represent a population, which has experienced sudden expansion, whereas high values of the raggedness index suggest stationary or bottlenecked populations. Second, we calculated *Fs* statistic of Fu (1997) using ARLEQUIN 3.1 (Excoffier et al. 2005), which is based on the probability of having a number of haplotypes greater or equal to the observed number of samples drawn from a constant-sized population. The significance of *Fs* was assessed with coalescent simulations.

### Estimations of divergence times

For testing whether genetic differentiation is due to pre-Quaternary and/or the Quaternary events, we calculated the divergence time of all haplotypes of *F. lucida* and *F. longipetiolata*, together with the haplotypes of *F. engleriana* and *F. hayatae* reported in Lei et al. (2012), using a Bayesian approach as implemented in beast 1.7.4 (Drummond and Rambaut 2007). Because a fragment from position 1102 to 1246 could not be aligned reliably, it was excluded from molecular dating analyses. Representatives of *Castanea*, *Lithocarpus,* and *Castanopsis* (Fagaceae) were used as functional outgroups. BEAST 1.7.4 has three substitution models (HKY, GTR, and TN93) and three site heterogeneity models (gamma, invariant sites, or gamma + invariant sites). We used GTR + G based on the result of AIC from jModelTest (Posada 2008) for this analysis under an uncorrelated lognormal relaxed clock model. We constrained all haplotypes of beeches to be monophyletic. The coalescent with constant size was used to model the tree prior. The stem group ages of *Fagus*, *Castanea*, and *Lithocarpus* were determined, respectively, as 47.0–53.0 Ma (Fossil taxon: *Fagus langevinii* Manchester and Dilhoff; Organ: cupules/nuts, leaves, pollen), 37.2–52.3 Ma (Fossil taxon: *Castanopsoidea columbiana* Crepet and Nixon; Organ: Pistillate inflorescences, fruits, pollen) and 23.0–33.9 Ma (Fossil taxon: *Lithocarpus saxonicus* H. Walther and Kvaček; Organ: Leaves with cuticle), and normal distribution priors were set for these calibration points. The detailed justification for each calibration point is provided by Sauquet et al. (2012): Table 2 and appendix S2). For each BEAST analysis, MCMC runs were performed, each of 1 × 10^7^ generations, with sampling every 1000 generations, following a burn-in of the initial 10% cycles. MCMC samples were inspected in TRACER 1.5 (http://beast.bio.ed.ac.uk/Tracer) to confirm sampling adequacy and convergence of the chains to stationary distribution. The effective sample size (ESS) of parameters was used to examine the mixing of the chains. Resulting chronograms were visualized in FIGTREE 1.3.1 (http://tree.bio.ed.ac.uk/software/figtree).

## Results

### Chloroplast haplotype variation, network and distribution

The length of the two combined chloroplast intergenic spacers (*atp*I-*atp*H and *ndh*J-*trn*F) was 1918 bp. Twenty-five substitutions and two indels (of 10 bp) were detected. A total of 25 haplotypes were characterized by 21 substitutions in four Chinese beeches (*F. lucida*, *F. longipetiolata*, *F. engleriana*, and *F. hayatae*; Table S2). Among them, twenty-three were found in *F. lucida* and *F. longipetiolata* (H1–H23). The two species shared four haplotypes with *F. engleriana* and *F. hayatae* [H17, H18, H19, and H20, corresponding to H1, H2, H4, and H5 in Lei et al. (2012), respectively]. H24 and H25 were specific to *F. engleriana* (Fig. 1A), which were coded as H3 and H6 in the previous study. *Fagus lucida* and *F. longipetiolata* possessed the same number of haplotypes (14 haplotypes, Table 1). Five haplotypes (H11, H12, H17, H19, and H20) were shared by the two species (Table 1, Figs. 1A and Fig. S2), *F. lucida* had nine private haplotypes (H1–H8, and H21), and *F. longipetiolata* harbored eight private ones (H9, H10, H13–H16, H22, and H23).

There was little taxonomic structure found in the haplotype network of the four Chinese beeches (Fig. 1A). Two haplotypes, H17 and H19, occurring at the highest frequencies across Chinese beeches, were internal to most of the other haplotypes. Four of six internal haplotypes, H11, H20, H17, and H24, linked to each other by one mutation and formed a loop. Interestingly, the tips on the network were mostly species-specific except for H12 and H18, and most tips connected to the internal nodes by just one step, with the exception of H1 and H2 connecting to H17 by three and two steps, respectively.

There were no obvious geographic structures in the haplotype distribution of *F. lucida* (Fig. 1B) and *F. longipetiolata* (Fig. 1C). Each species exhibited a mosaic distribution of haplotypes but little congruence between them. Within *F. lucida*, only H17 was widespread across its range, whereas H19 occurred in three geographically remote populations (BSZ in the easternmost part, and NJ and BJ in the westernmost part), and H4 was also present in relatively distant populations (CB, BDGS, NY, and TLB). Other *F. lucida* haplotypes were restricted to one or two populations. In addition, more than half of the populations (12 of 21) were polymorphic, and ancestral haplotypes H17 and/or H19 occurred in 11 of the polymorphic populations. In *F. longipetiolata*, range-wide distributed haplotypes were not detected. Most populations (25 of 28) were monomorphic, and population-specific haplotypes were also common in *F. longipetiolata* (e.g., H9, H13, H16, H22, and H23). The only haplotypes relatively widespread were H18 (shared by YJS, MES, and XC) and H20 (occurring in BSZ, WGS, LGS, and KKS), whereas two regionally widespread haplotypes (H17 in the central part and H19 in the northwestern part) were found.

Among the haplotypes shared by *F. lucida* and *F. longipetiolata* (Fig. S1), H17 was the most widespread and occurred across the whole range. The second most widespread haplotype was H20, which was scatteredly distributed from middle-west to the easternmost species' ranges. H11 and H12 were restricted to central areas, and H19 was disjunctly distributed in western China and in BSZ population, the eastern tip of both species' ranges. Regarding the haplotype frequency in the co-occurring studied locations of the two beeches, all population pairs but one (NL) possessed different haplotypes or, if shared, they were ancestral haplotypes (H17 and H20, Fig. S2).

### Genetic diversity and genetic structure

Total genetic diversity (*h*_T_) was slightly lower in *F. lucida* (0.887 ± 0.047) than in *F. longipetiolata* (0.927 ± 0.020), but within-population diversity (*h*_S_) of the former (0.262 ± 0.058) was much higher than that of the latter (0.060 ± 0.033). Consequently, genetic differentiation in *F. lucida* (*G*_ST_ = 0.705 ± 0.067; *N*_ST_ = 0.790 ± 0.086) was much lower than that in *F. longipetiolata* (*G*_ST_ = 0.936 ± 0.036; *N*_ST_ = 0.926 ± 0.042). Permutation tests indicated that *N*_ST_ was not significantly higher than *G*_ST_ both in *F. lucida* (*U* = 0.78, *P* > 0.05) and *F. longipetiolata* (*U* = −0.77, *P* > 0.05). Consistent with the mosaic distribution of haplotypes, SAMOVA analyses indicated that *F*_CT_ increased gradually with *K* values and reached a maximum value at *K* = 18 (Fig. S3) in *F. lucida*, which means that almost every population constituted a group. In *F. longipetiolata*, *F*_CT_ reached a submaximum value at *K* = 11, and fluctuated irregularly afterward (Fig. S3). When the two beeches in question were considered simultaneously, the AMOVA indicated that 5.14% of the total genetic variance was due to differences between species. The results of IBD analyses showed that genetic differentiation increased slowly with geographic distance in *F. longipetiolata* (*R*^2^ = 0.0265, *P* = 0.03; Fig. S4b), although no correlation between genetic differentiation and geographic distance was found in *F. lucida* (*R*^2^ = 0.0026, *P* = 0.46; Fig. S4a).

### Historical demography

The mismatch distributions for chlorotypes of *F. lucida* and *F. longipetiolata* were unimodal (data not shown). A low and nonsignificant raggedness value (*r* = 0.039, *P* = 0.55) in *F. lucida* suggests that the species might conform to the model of sudden population expansion. A significant raggedness value (*r* = 0.074, *P* < 0.001) found in *F. longipetiolata* suggests that this beech, instead, might have not experienced a sudden population expansion. Fu's *Fs* is considered to be more sensitive in detecting population expansion (Ramos-Onsins and Rozas 2002); values for *F. lucida* (*Fs* = −1.686; *P* = 0.065) and *F. longipetiolata* (*Fs* = −1.970; *P* = 0.054) indicated, however, that both beeches did not experience a recent demographic expansion. The historical demography was also inferred using the Bayesian skyline plot (Drummond et al. 2005) by estimating fluctuations in the effective population size. The results showed a recent population decline after long-term constant population size (data not shown), but the effective population size (ESS) was low possibly due to the low resolution of chloroplast sequences. Combining these data, we can assume that recent population expansion for both beeches is weak.

### Molecular dating

**Figure 2:**
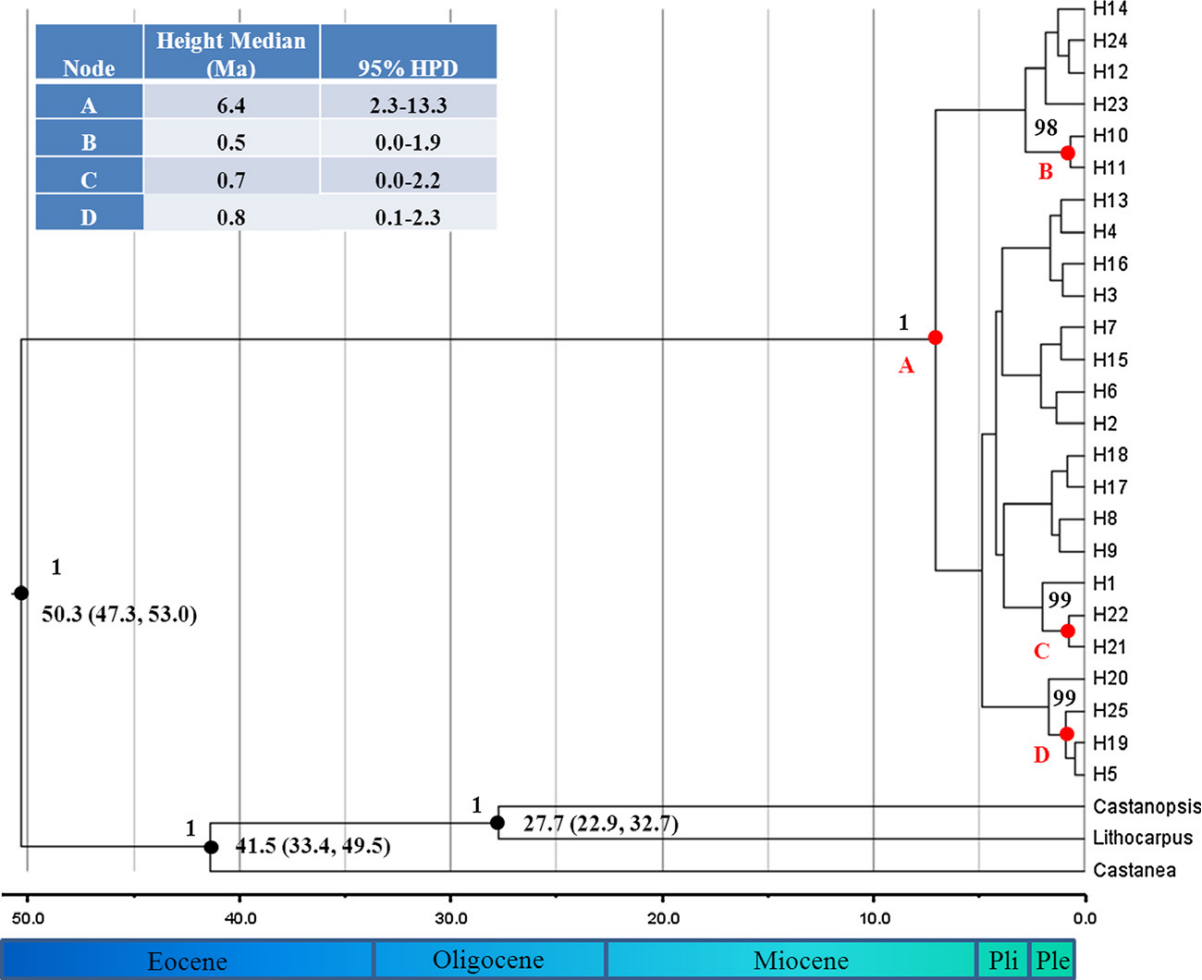
Chronograms of all haplotypes of four Chinese *Fagus* species (*F. lucida*, *F. longipetiolata*, *F. engleriana*, and *F. hayatae*) along with three genera of Fagaceae based on a Bayesian approach. Calibration points (closed black circles) are described in Sauquet et al. (2012). Numbers on the branches are posterior probabilities (PP), and the divergence time (in Ma) and 95% highest probability density (HPD) for the clades with above 95% PP supports are given in the up left table. Pli, Pliocene; Ple, Pleistocene.

The aligned sequence for constructing phylogeny of Chinese beeches' haplotype and three genera within Fagaceae was 1918 bp in length. Because a fragment from position 1102 to 1246 could not be aligned reliably, it was excluded from molecular dating analyses. The effective sample sizes exceeded 1000 for most parameters, suggesting well mixing of the chains. The time to the most recent common ancestor (TMRCA) was estimated to 6.38 Ma (95% HPD 2.29−13.25 Ma). Lineages started to diversify during the Pliocene; most haplotypes, however, diverged from each other during the Quaternary (2.49–0.50 Ma) (Fig. 2).

## Discussion

### The sharing of haplotypes between *F. lucida* and *F. longipetiolata*

Extensive sharing of cpDNA haplotypes among species had been observed in numerous woody genera (e.g., Palmé et al. 2004; Saeki et al. 2011). The correct interpretation of intraspecific phylogeographic data heavily relies on discriminating the source of haplotype sharing, that is, hybridization vs. incomplete sorting of ancient polymorphisms (Guicking et al. 2011), because the former can lead to erroneous phylogeographic hypotheses for the studied species (Saeki et al. 2011).

Several lines of evidence suggest that incomplete sorting seems to be the better explanation for the sharing of haplotypes between *F. lucida* and *F. longipetiolata*, although the possibility of hybridization cannot be excluded. First, in the Miocene, *Fagus* is found throughout the Northern Hemisphere up to very high latitudes and characterized by unhindered gene flow among species (Denk and Grimm 2009). The large population size and ancient gene flow within progenitors could be the explanation for sharing of haplotypes between two distinct-related Japanese beeches, *F. crenana* and *F. japonica* (Okaura and Harada 2002), as well as the pattern observed in this study. Second, 17 of 23 haplotypes are confined to a single species (*F. lucida* and *F. longipetiolata* have nine and eight private haplotypes, respectively) and the few shared haplotypes are mainly restricted to the central part of the network where the most ancient alleles are supposed to occur (Crandall and Templeton 1993). Even taking *F. engleriana* and *F. hayatae* into consideration, this pattern remains unchanged (Fig. 1a). Moreover, all shared haplotypes are largely widespread and often occur in nonoverlapping areas (Fig. S1), further suggestive of the antiquity of the shared variants (Jakob and Blattner 2006). Third, the generally low numbers of missing intermediate haplotypes found in this study (Fig. 1a) indicate low extinction rates and long-term persistence of chloroplast lineages within Chinese beeches, which would have resulted in low rates of lineage sorting. This phenomenon is frequent for woody species with large population sizes and growing in climatically stable regions (Cannon and Manos 2003; Guicking et al. 2011). Fourth, the two beeches inhabit some mixed stands (Fig. S2), which provides ample opportunities for hybridization. However, all shared haplotypes—as explained above—are putatively ancestral lineages and most populations in mixed stands possess different haplotypes, suggesting that hybridization between *F. lucida* and *F. longipetiolata* is infrequent. Low rates hybridization can also be deduced from the strikingly morphological differences that exist between the two beeches (Huang et al. 1999; Denk 2003). In addition, AMOVA analysis indicates that a considerable amount of variance (5.14%) is attributable to among-species differences, which is in contrast to some examples where hybridization among species prevails and *F*_CT_ values were reported. For example, in the three main *Betula* species in Europe (*Betula pendula*, *B. pubescens,* and *B. nana*), *F*_CT_ is not significantly different from zero (Palmé et al. 2004).

### Evolutionary history of the two Chinese beeches

Taking advantage of the well-studied fossil record in Fagaceae, we estimated the age of crown group of Chinese beeches to late Miocene (6.36 Ma). Lineages started to diversify around the Miocene–Pliocene boundary, and this process would have continued during the Pliocene and intensified during the Pleistocene: the estimated age of all the recovered haplotypes of Chinese beeches is younger than 2.6 Mya (Fig. 2). These time estimates are exactly consistent with the crown age of another relict genus present in subtropical China, *Cercidiphyllum* (*ca*. 6.52 Ma, HPD: 1.34–13.42) (Qi et al. 2012), and the time of haplotype divergence within *Cercidiphyllum* (Qi et al. 2012). Another relict species from subtropical China, *Taiwania cryptomerioides*, also show haplotype diversification beginning from pre-Quaternary times (3.4–3.2 Ma; Chou et al. 2011). These results confirm that the evolutionary history of Cenozoic relict taxa in subtropical China was deeply impacted by the climatic and geological changes since the Neogene, stressing the need for considering both pre-Quaternary and the Quaternary events in the phylogeographic studies of subtropical organisms in China.

According to a biogeographic analysis based on extensive fossil records (Denk and Grimm 2009)*, Fagus* evolved in the Northern Pacific region in the middle Eocene and was found throughout the Northern Hemisphere up to very high latitudes (e.g., Iceland, Alaska) in the Miocene. In post-Miocene times, *Fagus* underwent range reduction and/or migration toward the south, coupled with extinction from the northernmost latitudes, western North America and Central Asia (Denk and Grimm 2009). According to these authors, the modern species and their ranges were progressively shaped during the Pliocene and the Quaternary. Subtropical China is regarded as the extant center of diversity for *Fagus* (Shen 1992), as it harbors about half of all *Fagus* taxa. This current high diversity should be explained by several late Neogene migrations from Central Asia and northeastern Asia. However, the exact timing of beeches' migration to subtropical China (and adjacent Northern Vietnam and Taiwan) is uncertain (Denk and Grimm 2009). The crown group age (6.36 Ma) of Chinese beeches estimated in this study suggests that the migration event could have happened by the time of Miocene–Pliocene boundary, triggered by a series of climatic and geologic events. According to high-resolution deep-sea oxygen (δ^18^O) and carbon (δ^13^C) isotope records, the Cenozoic global cooling, which resumed after the Mid-Miocene Climate Optimum (*ca*. 15 Ma), experienced an additional cooling and a small-scale ice-sheet expansion by the late Miocene–early Pliocene, *ca*. 6–7 Mya (Zachos et al. 2001). The global cooling, coupled with the significant uplift of Tibetan–Himalayan Plateau during the late Miocene (8–10 Ma, An et al. 2001), drove late Cenozoic stepwise aridification in Central Asia (including northern China), with a peak just before the Miocene–Pliocene boundary (8–7 Ma, Guo et al. 2002). Meanwhile, and closely linked with global cooling and the uplift of Tibetan–Himalayan Plateau, Indian and East Asian monsoons were formed (or significantly intensified) about 8–9 Ma (An et al. 2001; Zheng et al. 2004). Therefore, since the late Miocene–early Pliocene onward, the southward migration of the ancestors of Chinese beeches could have been triggered by the global cooling and aridification in Central Asia, and the establishment of monsoon climate in subtropical China would have provided a major shelter for those ancestors throughout the Quaternary glacial/interglacial cycles.

A large body of literature has documented the influence of the Quaternary climate changes on population genetic structure of plant species in subtropical China (reviewed in Qiu et al. 2011; Liu et al. 2012 and references therein). The fact that most haplotypes are dated to be younger than 2.6 Ma indicates that the Quaternary climate changes must have played a very important role in the population differentiation of Chinese beeches. Moreover, we found plenty of private haplotypes within populations (Fig. 1B,C) and weak signs of recent population expansion for both *F. lucida* and *F. longipetiolata*, confirming the emerging phylogeographic pattern of multiple glacial refugia and little inter-/postglacial expansion for subtropical plants of China (Qiu et al. 2011; Lei et al. 2012; Liu et al. 2012). This kind of phylogeographic structure matches well with the isolated distribution pattern of Chinese beeches (Liu et al. 2003; Fang and Lechowicz 2006; Guo and Werger 2010) and is also supported by fossil records in China. Pollen records indicate that *Fagus* occurred across subtropical China during the Quaternary, but generally scatteredly in mountainous areas and always showing limited expansions (Liu et al. 2003). It is most likely that the two beeches would have colonized the whole subtropical China (including Northern Vietnam and Taiwan) before the Quaternary, when the climate showed less seasonality and was more humid (Axelrod et al. 1996; An et al. 2001); during the Quaternary, these beeches were forced to retreat to mountain areas, the only places with enough humidity and ecological amplitude to track the climatic changes (Liu et al. 2003). At glacial periods, beech expansions into the lowlands were forcibly limited because the lack of enough moisture in the lowland. At the interglacials—such as the Holocene—its development at low elevations was avoided by the expansion of evergreen broad-leaved forests (Liu et al. 2003; Fang and Lechowicz 2006; Lei et al. 2012). Because the major increase in aridification after 3.5 Ma in Central Asia (Guo et al. 2002), colonization of high latitudes in the North during interglacials was also unlikely for Chinese beeches, which has happened for beech species in Europe, North America, and Japan (Okaura and Harada 2002; Magri et al. 2006; Morris et al. 2010).

### Species-specific mosaic distribution of haplotypes in *F. lucida* and *F. longipetiolata*

The main goal of comparative phylogeography has been to search for concordant geographic distribution among lineages within different species, which would indicate the influence of common historical factors (Michaux et al. 2005). Given their phylogenetic and ecological similarities, sympatric congeneric species might be more likely to display congruent phylogeographic patterns (Garrick et al. 2008). In the present study, we found that haplotype distribution exhibited species-specific geographic mosaic patterns for *F. lucida* and *F. longipetiolata,* that is, mosaic distribution within each species but lack of concordance between them (Fig. 1B,C). Such kind of phylogeographic pattern has been observed in subtropical China (see reviews in Qiu et al. 2011 and Liu et al. 2012), as well as other regions in the world, including Southeast Asia (Cannon and Manos 2003), southern Australia (Byrne 2008), South America (Turchetto-Zolet et al. 2012), southern Europe (Nieto-Feliner 2011), and even in very small territories such as the island of Corsica (north-western Mediterranean Basin, Bisconti et al. 2013). However, the present study reports for the first time a pattern of species-specific geographic mosaic of lineages in sympatric congeneric species in subtropical China. The findings of this study strengthen the belief that historical population dynamics in this area is extremely complex (Qiu et al. 2011) and points out the need for more comparative studies to fill the knowledge gaps in the evolutionary history of the rich flora of subtropical China.

There are two possible explanations for the formation of geographic mosaic patterns of lineages in both beech species: founder effects due to long-distance dispersal (Petit et al. 1997) or long-term isolation among mosaic habitats (Särkinen et al. 2012). Whereas the consequence of the former model is usually a patchy distribution of the haplotypes at a regional scale (Petit et al. 1997), a range-wide mosaic of lineages is expected for the latter (Särkinen et al. 2012). Therefore, the range-wide geographic mosaic of Quaternary lineages observed in *F. lucida* and *F. longipetiolata* is more likely to result from in situ diversification in the mosaic habitats. Ecological studies indicate that beeches can only grow on places with a cool and moist climate and have a narrow ecological niche (Guo and Werger 2010). These characteristics determine that beeches in China are restricted to subtropical mountain areas while their populations are isolated by the extensive evergreen broad-leaved forests of the lowlands (Fang and Lechowicz 2006). With this distribution pattern, gene flow by seeds among populations is difficult, and stochastic factors such as genetic drift within isolated populations would thus have had a prevailing role on the evolutionary processes. Such a scenario would have lead to a mosaic distribution of haplotypes and lack of phylogeographic congruence among species (Fig. 1B,C). Because migration cannot counteract the effects of genetic drift, genetic distance is not correlated with geographic distance in *F. lucida*, and only weakly correlated with *F. longipetiolata* (Fig. S4a,b Hutchison and Templeton 1999). The results of SAMOVA analyses show that *K* values are very high in both *F. lucida* and *F. longipetiolata*, further supporting that there are significant genetic barriers among populations (Dupanloup et al. 2002).

Apart from the species-specific distribution of haplotypes, other differences between the phylogeographic structure of *F. lucida* and *F. longipetiolata* are worth noting. First, there are two widely distributed haplotypes found in the former (Fig. 1B), but not in the latter (Fig. 1C), which is responsible for the lower genetic differentiation in *F. lucida*. Second, much higher within-population genetic diversity was found in *F. lucida* than in *F. longipetiolata*. Given the similar life history and breeding system in beeches (i.e., with the same potential for gene flow), contrasting levels of intrapopulation diversity between the two beeches can only be explained by differences in their population size. Indeed, *Fagus lucida* has a wider ecological niche and always maintains larger populations (Z.-Y. Zhang, pers. obs.) than *F. longipetiolata*. In large populations, high genetic diversity is generally expected, and ancestral haplotypes are more likely to be retained (Ellegren 2009). Therefore, lower genetic differentiation and more widespread ancestral lineages in *F. lucida* may be a consequence of historical rather than contemporary gene flow. However, data obtained from this study do not allow us to distinguish between historical and contemporary gene flow; thus, more efforts (e.g., parentage analysis) are needed to make direct comparisons of gene flow between *F. lucida* and *F. longipetiolata*.

## Supporting Information

**Table S1.** The outgroups used in molecular dating analysis.

**Table S2.** Substitutions of the aligned sequences of two chloroplast DNA fragments.

**Figure S1.** The distribution of shared haplotypes.

**Figure S2.** The haplotype frequency in the co-occurring locations.

**Figure S3.** The values of genetic variance (*F*_CT_) in SAMOVA analysis.

**Figure S4.** Correlation between genetic distances (*F*_ST_) and geographic distances.
