# Supplementary material for: Comparative phylogeography of two sympatric beeches in subtropical China: Species-specific geographic mosaic of lineages
Source: Ecol Evol. 2013 Oct 11;3(13):4461–72. doi: 10.1002/ece3.829 (PMC3856746; doi:10.1002/ece3.829)
Supplement: Supplementary file 1 [file ece30003-4461-SD1.docx]

**Table S1** Plant species and voucher information for the outgroups used in molecular dating analysis

| Species | voucher | GenBank accession numbers  atpI-atpH ndhJ-TabE | |
| --- | --- | --- | --- |
| *Castanea henryi* | Wurong 1201 (JXAU) | KC797393 | KC905025 |
| *Castanopsis tibetana* | Wurong 1202 (JXAU) | KC797395 | KC797387 |
| *Lithocarpus glaber* | Wurong 1203 (JXAU) | KC797394 | KC797388 |

**Table S2** Substitutions of the aligned sequences of two chloroplast DNA fragments in 25 haplotypes of four Chinese beeches (*F. lucida*, *F. longipetiolata*, *F. engleriana*, and *F. hayatae*) and the GenBank accession number (GB No.) for each sequence.

| Nucleotide position | *atp*I-*atp*H | | | | | | | | | | | |  | GB No. | *ndh*J-*trn*F | | | | | | | | | |  |  | GB No. |
| --- | --- | --- | --- | --- | --- | --- | --- | --- | --- | --- | --- | --- | --- | --- | --- | --- | --- | --- | --- | --- | --- | --- | --- | --- | --- | --- | --- |
|  | 3 | 1 | 2 | 3 | 3 | 3 | 3 | 5 | 5 | 6 | 8 | 9 | 9 |  | 3 | 1 | 1 | 1 | 2 | 3 | 3 | 4 | 5 | 5 | 6 | 8 |  |
|  | 5 | 7 | 7 | 0 | 2 | 5 | 7 | 2 | 9 | 3 | 0 | 6 | 8 |  |  | 8 | 0 | 7 | 8 | 3 | 6 | 3 | 7 | 8 | 7 | 8 |  |
|  |  | 9 | 8 | 7 | 8 | 4 | 6 | 0 | 3 | 1 | 8 | 9 | 1 |  |  |  | 6 | 1 | 2 | 6 | 2 | 9 | 6 | 5 | 3 | 7 |  |
| Hap 1 | T | C | C | C | T | A | T | G | A | G | G | C | G | KC853723 | G | G | A | G | A | T | G | A | C | A | C | T | KC853742 |
| Hap 2 | T | C | A | C | G | A | T | G | A | G | G | G | G | KC853724 | G | A | A | A | A | T | G | A | C | A | C | T | KC853731 |
| Hap 3 | T | C | C | C | T | A | T | G | A | G | G | G | G | KC853726 | G | A | A | A | A | T | G | G | C | G | C | T | KC853733 |
| Hap 4 | T | C | C | C | T | A | T | G | A | G | G | G | T | KC853727 | G | A | A | A | A | T | G | A | C | A | C | T | KC853731 |
| Hap 5 | T | C | C | C | T | A | T | G | A | G | G | G | T | KC853727 | T | A | A | A | A | T | G | A | T | A | C | T | KC853736 |
| Hap 6 | T | C | C | C | T | A | T | G | A | T | G | G | G | KC853729 | G | A | A | A | A | T | G | A | C | A | C | T | KC853731 |
| Hap 7 | T | C | C | C | T | A | T | G | A | G | G | G | G | KC853730 | G | A | A | A | A | T | A | A | C | A | C | T | KC853741 |
| Hap 8 | T | C | C | C | T | G | T | G | A | G | G | G | G | KC876055 | G | A | A | A | A | T | G | A | C | A | C | T | KC853731 |
| Hap 9 | T | C | C | C | T | A | T | G | A | G | G | G | G | JX179091 | G | A | A | A | A | T | G | A | C | A | C | T | JX179108 |
| Hap 10 | C | A | C | C | T | A | T | G | A | G | G | G | G | JX179081 | T | A | A | A | A | T | G | A | C | A | C | C | JX179096 |
| Hap 11 | T | A | C | C | T | A | T | G | A | G | G | G | G | KC853725 | T | A | A | A | A | T | G | A | C | A | C | T | KC853732 |
| Hap 12 | T | A | C | C | T | A | T | G | C | G | G | G | G | KC853728 | G | A | A | A | A | T | G | A | C | A | C | T | KC853731 |
| Hap 13 | T | C | C | C | T | A | T | G | A | G | G | G | G | JX179077 | G | A | A | A | A | A | G | A | C | A | C | T | JX179093 |
| Hap 14 | T | A | C | C | T | A | T | G | A | G | G | G | G | JX179075 | G | A | A | A | A | T | G | A | C | A | T | T | JX179106 |
| Hap 15 | T | C | C | C | T | A | T | G | A | G | A | G | G | JX179085 | G | A | A | A | A | T | G | A | C | A | C | T | JX179107 |
| Hap 16 | T | C | C | C | T | A | T | G | A | G | G | G | G | JX179088 | G | A | G | A | A | T | G | A | C | A | C | T | JX179105 |
| Hap 17 | T | C | C | C | T | A | T | G | A | G | G | G | G | KC853726 | G | A | A | A | A | T | G | A | C | A | C | T | KC853731 |
| Hap 18 | T | C | C | C | T | A | C | G | A | G | G | G | G | JX179086 | G | A | A | A | A | T | G | A | C | A | C | T | JX179107 |
| Hap 19 | T | C | C | C | T | A | T | G | A | G | G | G | G | KC853726 | T | A | A | A | A | T | G | A | T | A | C | T | KC853736 |
| Hap 20 | T | C | C | C | T | A | T | G | A | G | G | G | G | KC853726 | T | A | A | A | A | T | G | A | C | A | C | T | KC853735 |
| Hap 21 | T | C | C | T | T | A | T | G | A | G | G | G | G | KC797282 | G | A | A | A | G | T | G | A | C | A | C | T | KC797332 |
| Hap 22 | T | C | C | C | T | A | T | G | A | G | G | G | G | KC797231 | G | A | A | A | G | T | G | A | C | A | C | T | KC797340 |
| Hap 23 | T | A | C | C | T | A | T | A | A | G | G | G | G | KC797276 | G | A | A | A | A | T | G | A | C | A | C | T | KC797377 |
| Hap 24 | T | A | C | C | T | A | T | G | A | G | G | G | G | HM347325 | G | A | A | A | A | T | G | A | C | A | C | T | HM347326 |
| Hap 25 | T | C | C | C | T | A | C | G | A | G | G | G | G | HM347324 | T | A | A | A | A | T | G | A | T | A | C | T | HM347327 |


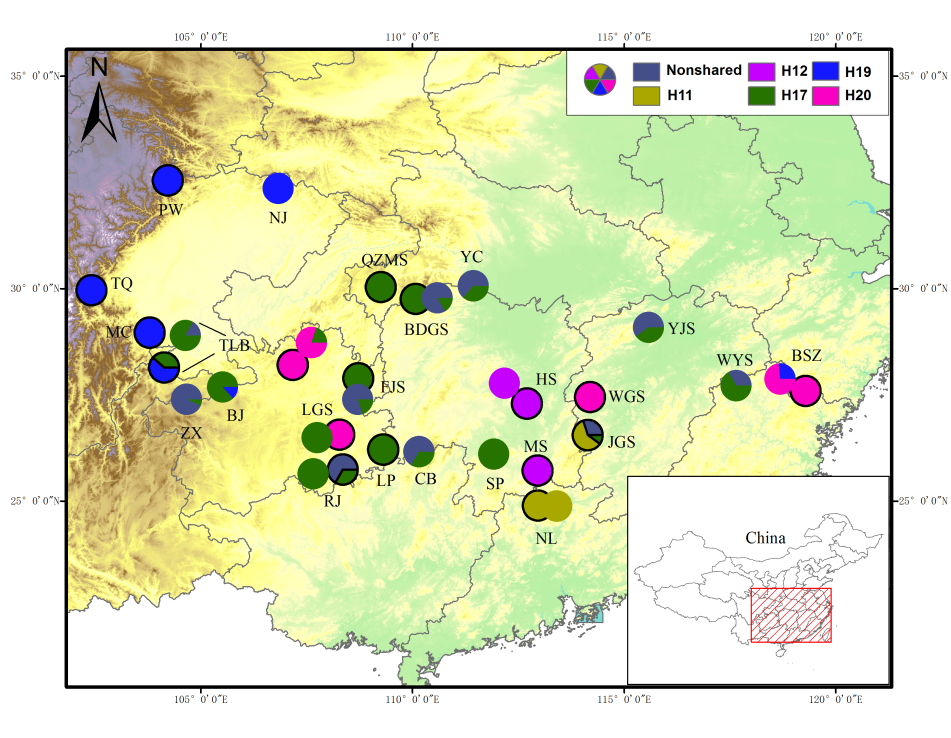


**Figure S1** The distribution of shared haplotypes between *F. lucida* and *F. longipetiolata* (i.e., H11, H12, H17, H19 and H20). Cycles without black edge: *F. lucida*, cycles with black edge: *F.* *longipetiolata*. Nonshared colour includes all the non-shared haplotypes that occur within populations that have shared haplotypes between the two species.


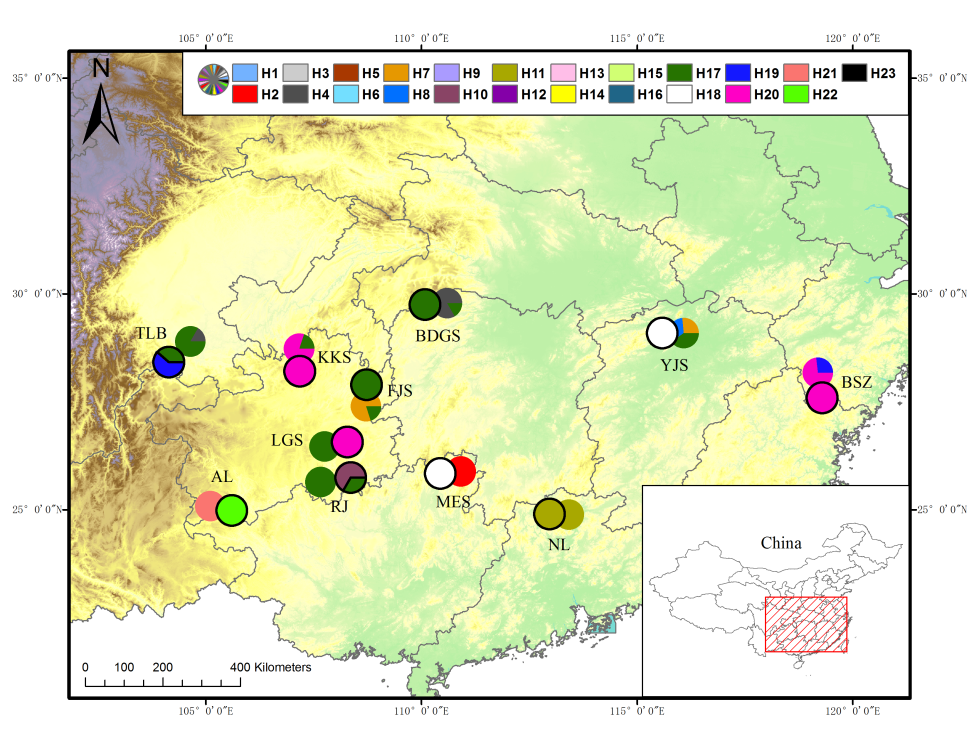


**Figure S2** The haplotype frequency of *F. lucida* and *F. longipetiolata* in the co-occurring locations. Cycles without black edge: *F. lucida*, cycles with black edge: *F.* *Longipetiolata*.


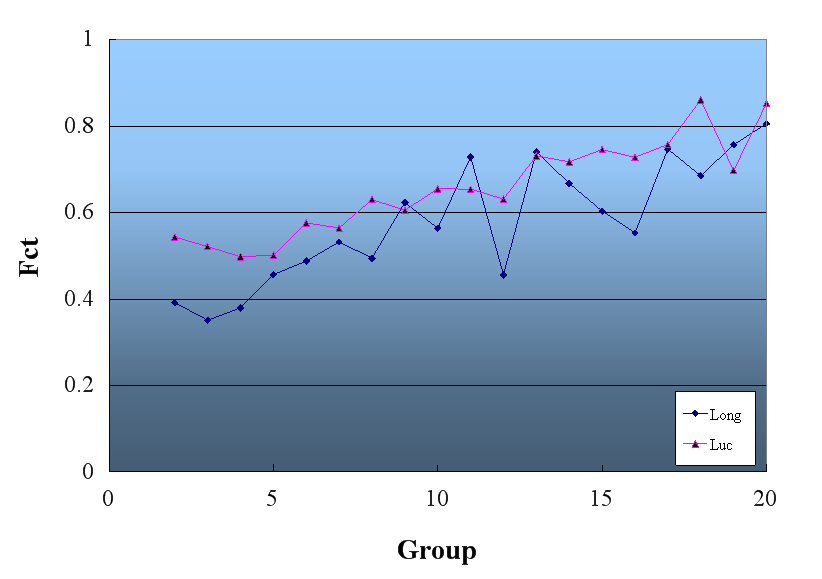


**Figure S3** The values of genetic variance (*F*_CT_) of *Fagus lucida* and *F. longipetiolat*a partitioned among different groups (*K*) in SAMOVA analysis


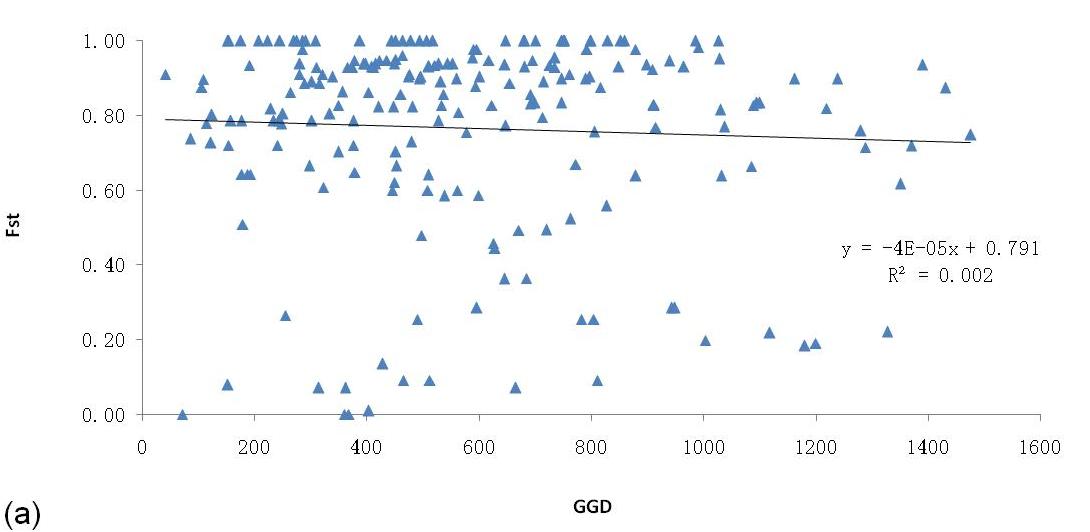

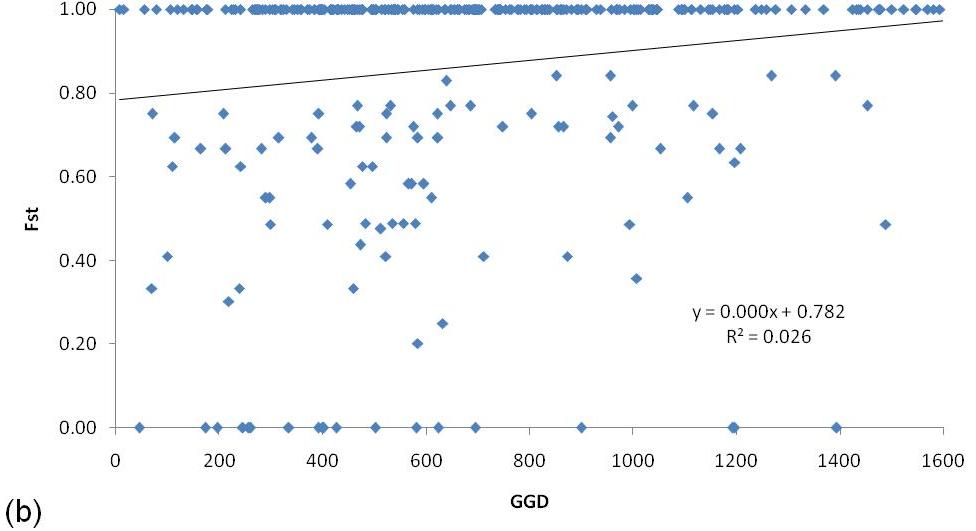


**Figure S4** Correlation between genetic distances (*F*_ST_) and geographical distances (kilometres) separating each pairwise combination of populations within *Fagus lucida* (*P* = 0.46, a) and *F. longipetiolata* (*P* = 0.03, b)
